# Early Longitudinal Changes in Radiomic Tumor Heterogeneity Predict Progression-Free Survival in Advanced Non-Small Cell Lung Cancer

**DOI:** 10.3390/cancers18183033

**Published:** 2026-09-18

**Authors:** Zachary Thompson, Junmin Whiting, Olyo Stringfield, Mahmoud Abdalah, Sebastian Viracacha, Jhanelle Gray, Andreas Saltos, Dung-Tsa Chen

**Affiliations:** 1 Biostatistics and Bioinformatics Department, Moffitt Cancer Center, Tampa, FL 33612, USA; zachary.thompson@moffitt.org (Z.T.); junmin.whiting@moffitt.org (J.W.); 2 Quantitative Imaging Shared Resource, Moffitt Cancer Center, Tampa, FL 33612, USA; olya.stringfield@moffitt.org (O.S.); mahmoud.abdalah@moffitt.org (M.A.); 3 Department of Thoracic Oncology, Moffitt Cancer Center, Tampa, FL 33612, USA; sebastian.viracacha@moffitt.org (S.V.); jhanelle.gray@moffitt.org (J.G.); andreas.saltos@moffitt.org (A.S.)

**Keywords:** radiomics, tumor heterogeneity, non-small cell lung cancer

## Abstract

Radiomic features derived from longitudinal imaging offer a non-invasive approach to quantify tumor heterogeneity, but their integration with clinical variables in small cohorts remains methodologically challenging. We analyzed 23 patients with advanced non-small cell lung cancer (NSCLC) enrolled in a Phase I study of pembrolizumab and vorinostat. Radiomic features were extracted from baseline and two-month follow-up computed tomography scans, aggregated to the patient level, and transformed into delta features representing early changes over time. A unified modeling framework was implemented using strict leave-one-out cross-validation (LOOCV), incorporating both radiomic and clinical variables. Within each training fold, outcome-guided feature screening was performed using elastic net penalized Cox regression, followed by category-aware principal component analysis and ridge-penalized Cox modeling. Model performance was evaluated using out-of-fold concordance indices for overall survival (OS) and progression-free survival (PFS). Radiomics-only models demonstrated moderate discrimination for OS (C-index 0.609) and favorable discrimination for PFS (C-index 0.770), whereas clinical-only models showed weaker performance (OS 0.605; PFS 0.571). The combined radiomics and clinical model demonstrated numerically higher discrimination without statistically distinguishable differences for OS (C-index 0.648) while maintaining favorable discrimination for PFS (C-index 0.718). Stable features were predominantly texture-based and included Laws filter and co-occurrence-derived metrics associated with spatial heterogeneity. In this exploratory analysis, early changes in radiomic features reflecting tumor heterogeneity suggest potential associations with survival outcomes in advanced NSCLC. Integration of radiomic and clinical variables showed trends toward improved performance for overall survival while maintaining favorable discrimination for progression-free survival. These findings support further investigation of longitudinal radiomic features as candidate imaging biomarkers in larger, independently validated cohorts.

## Simple Summary

Medical imaging can reveal microscopic tumor patterns that may predict treatment response. In this study, we investigated whether early changes in these patterns—called radiomic features—extracted from computed tomography scans before and two months into therapy could predict survival in 23 patients with advanced non-small cell lung cancer receiving pembrolizumab and vorinostat. Using a rigorous cross-validation framework suited to small cohorts, we found that texture-based radiomic features capturing spatial tumor heterogeneity were strongly associated with progression-free survival. Radiomic models substantially outperformed clinical-only models for progression-free survival, and combining radiomic and clinical variables showed trends toward improved discrimination for overall survival. These results suggest that longitudinal radiomic features reflecting early tumor changes may serve as candidate imaging biomarkers, warranting validation in larger, independent cohorts.

## 1. Introduction

Tumor heterogeneity is a fundamental driver of treatment resistance and clinical outcomes in cancer, reflecting spatial and temporal variation in tumor cell populations and microenvironmental conditions [1,2]. In advanced non-small cell lung cancer (NSCLC), intratumoral heterogeneity underlies variability in treatment response and disease progression, ultimately contributing to differences in survival outcomes.

Radiomics enables high-throughput extraction of quantitative features from medical imaging, providing a non-invasive approach to characterize tumor phenotype beyond conventional size-based metrics [3]. Prior work has demonstrated that radiomic signatures can capture biologically relevant information related to tumor heterogeneity and are associated with clinical outcomes across multiple cancer types [4]. In particular, texture-based features derived from gray-level co-occurrence matrices (GLCMs), run-length matrices, and filter-based methods such as Laws energy filters quantify spatial organization and intensity variation within tumors, which may reflect underlying tumor biology and microenvironmental characteristics [3,5,6].

Despite these advances, several challenges limit the clinical application of radiomics. High-dimensional feature spaces, strong inter-feature correlations, and small sample sizes increase the risk of overfitting and reduce model stability. Furthermore, radiomic features are often analyzed independently of clinical variables, which may provide complementary prognostic information.

Delta radiomics, defined as the change in radiomic features over time, has emerged as a promising approach to capture early treatment response and dynamic tumor behavior [7]. However, robust statistical frameworks that integrate longitudinal radiomics with clinical variables while maintaining strict validation are still needed.

In this study, we developed a unified modeling framework that integrates radiomic and clinical features within a strictly cross-validated pipeline. This approach combines outcome-guided feature selection, category-aware dimensionality reduction, and penalized survival modeling to generate out-of-fold predictions. We hypothesized that early changes in radiomic features reflecting tumor heterogeneity would be predictive of survival outcomes and that integration with clinical variables would improve predictive performance.

To our knowledge, this is among the first studies to integrate longitudinal radiomic features and clinical variables within a strictly cross-validated modeling framework in a small early-phase clinical cohort. The framework was specifically designed for small, high-dimensional clinical datasets by combining fold-specific feature screening, category-aware dimensionality reduction, and penalized survival modeling. Given the small sample size, the analysis is intended to be exploratory and hypothesis-generating.

## 2. Materials and Methods

### 2.1. Study Cohort and Data Preparation

A total of 120 patients were enrolled in a Phase I clinical trial of pembrolizumab and vorinostat for advanced NSCLC. Of these, 32 had evaluable tumor segmentations available, and 29 had both baseline and two-month follow-up computed tomography scans with complete radiomic extraction, enabling calculation of delta radiomic features. Radiomic features were extracted using standardized definitions to support reproducibility [6] from baseline and two-month follow-up computed tomography scans. For each lesion, radiomic features were calculated at both time points and then aggregated to the patient level by averaging values across all segmented lesions. This lesion-averaging approach was used to characterize the overall tumor burden and radiomic phenotype across disease sites, rather than relying on a single dominant lesion that may not adequately represent systemic disease biology. Delta radiomic features were defined as the difference between follow-up and baseline patient-level values, thereby capturing early changes in tumor phenotype. After preprocessing and merging the radiomic data with the clinical and survival datasets, 23 patients had complete radiomic and outcome information required for analysis. Six patients were excluded due to missing clinical outcome data—specifically survival time or vital status—that were required for merging the radiomic and survival datasets.

Because OS and PFS were defined at the patient level, lesion-level radiomic measurements were aggregated to produce one value per patient for each radiomic feature. Patients had a median of 3 lesions (mean 2.8; range 1–5). Feature values were averaged across all evaluable target lesions at each imaging time point before calculating longitudinal changes. This approach avoided treating multiple lesions from the same patient as independent observations, ensured that each patient contributed equally to the survival models, and provided a summary measure of the overall radiomic phenotype across disease sites. We recognize that averaging does not directly preserve the full extent of inter-lesion heterogeneity; therefore, the resulting variables should be interpreted as patient-level summaries of the average tumor phenotype rather than direct measures of lesion-to-lesion variability.

All CT images were contrast-enhanced standard-of-care imaging studies performed on Siemens and GE scanners. Imaging parameters followed clinical trial protocols, with slice thickness ranging from 3 mm to 5 mm and pixel spacing ranging from 0.55 mm to 0.98 mm. Image intensities were discretized using a fixed bin width of 25 Hounsfield units before texture-feature calculation. No voxel resampling, spatial interpolation, or scanner-specific harmonization was performed before radiomic feature extraction. Radiomic features were therefore calculated using the original image voxel dimensions and acquisition characteristics. Scanner vendor, slice thickness, and pixel spacing were not included as adjustment variables in the primary models.

Target solid tumors were evaluated by a radiologist according to RECIST 1.1. For each target lesion, the CT slice number and largest cross-sectional diameter were recorded in standardized tumor measurement worksheets (TMWs). An imaging scientist with 15 years of experience used the TMWs to identify all target lesions and perform volumetric segmentation using a single-click ensemble segmentation approach in HealthMyne version 13.3.13 [8]. Segmentation quality was evaluated through visual review of lesion coverage and contour placement, and approximately 10% of segmentations were randomly reviewed by a radiologist for quality assurance. Segmentations were performed once by a single observer; independent repeat segmentations by the same observer or by a second observer were not available. Consequently, formal intraobserver and interobserver reproducibility analyses were not performed. Final tumor segmentations were stored in RTSTRUCT format for subsequent radiomic analysis.

Radiomic features were extracted from the entire segmented tumor volumes using an in-house radiomics pipeline developed and validated in accordance with the Image Biomarker Standardization Initiative [6]. A total of 309 radiomic features were extracted from each lesion. These features included measures of volume, shape, first-order intensity, and second-order texture statistics.

Radiomic features were grouped into predefined categories based on extraction class, including shape, first-order intensity, and texture features such as GLCM, GLRLM (Gray-Level Run Length Matrix), and GLSZM (Gray-Level Size Zone Matrix), consistent with standardized radiomics frameworks [6]. Additional filter-based features, including Laws and wavelet-transformed features, were also derived.

To reduce the influence of skewness and differences in feature scale, radiomic features with substantial skewness were transformed using the Yeo–Johnson transformation [9] and then standardized using Z-scores.

The final analytic cohort included 23 patients with advanced non-small cell lung cancer who had complete radiomic, clinical, and survival data. All patients experienced events for both overall survival (OS) and progression-free survival (PFS), resulting in a 100% event rate for both endpoints. Therefore, follow-up time corresponded directly to observed event times. OS ranged from 1.8 to 74.9 months, with a median of 8.77 months, and PFS ranged from 0.3 to 45.5 months, with a median of 4.6 months. Progression-free survival was defined as the time from study entry to radiographic or clinical disease progression or death, consistent with standard RECIST-based assessment criteria. Clinical variables incorporated into the modeling framework included age at study entry and baseline disease-burden indicators, specifically the presence of lung lesions and lymph node involvement, each encoded as a binary variable.

All statistical analyses were performed using R version 4.5.3. The primary packages used were survival for survival outcomes and concordance calculations, glmnet for elastic net and ridge-penalized Cox regression, survminer for Kaplan–Meier visualization, and tidyverse, including dplyr, tidyr, purrr, and ggplot2, for data management and visualization; complete package versions are listed in Appendix B (Table A1).

### 2.2. Modeling Framework

A unified modeling framework was implemented using leave-one-out cross-validation (LOOCV) [10], ensuring that all preprocessing, feature selection, and model fitting steps were restricted to the training data within each fold to prevent information leakage. Separate models were constructed for overall survival (OS) and progression-free survival (PFS), with out-of-fold predictions generated for all patients.

All preprocessing, feature selection, and dimensionality reduction steps were conducted within each training fold to prevent information leakage. All 309 radiomic features were initially considered as candidate predictors. Within each LOOCV training fold, features with zero or nonfinite variance were excluded before elastic-net screening. Features with missing training-set values or zero variance were also excluded before category-specific PCA. No prior filtering based on test–retest reliability, segmentation perturbation, or intraclass correlation coefficients was performed because such reproducibility data were not available.

Within each LOOCV training fold, outcome-guided feature screening was performed using elastic net penalized Cox regression with a mixing parameter of α=0.5 [11]. The mixing parameter was fixed at α=0.5 to provide a balance between shrinkage and LASSO-type variable selection in the presence of highly correlated radiomic features. Sensitivity analyses were also conducted using α=0.25 and α=0.75 to evaluate the robustness of feature-selection frequencies to greater ridge-like and LASSO-like penalization, respectively.

Penalty parameters were selected via internal cross-validation. Features with non-zero coefficients at the one-standard-error threshold ($\lambda_{1se}$) were retained. If no features survived this threshold, selection fell back to the minimum-penalty solution ($\lambda_{\min}$); if that also yielded no features, the top *K* features by absolute coefficient magnitude at $\lambda_{\min}$ were taken (K=5). The final retained set was capped at K=5, ranked by absolute coefficient at $\lambda_{\min}$. This screening was performed independently for OS and PFS, and the union of both screens was carried forward into the category-aware PCA step. Across folds, this procedure typically resulted in the selection of approximately 7–10 radiomic features.

Selected features were mapped to predefined radiomic categories, and dimensionality reduction was performed using category-aware principal component analysis [12]. When multiple features were present within a category, the first principal component was retained, while single features were standardized and treated as one-dimensional components. This approach preserved the structural grouping of radiomic feature classes while controlling dimensionality. Across folds, this process yielded approximately 3–4 radiomic principal components per model.

Clinical variables were processed within each training fold, with continuous variables imputed using the median and categorical variables encoded using model matrices. Radiomic components and clinical variables were then combined into a unified predictor matrix.

Modeling was performed using a ridge-penalized Cox proportional hazards model [13] (α=0), with the penalty parameter selected via internal cross-validation using the minimum cross-validated error ($\lambda_{\min}$) [14,15]. Within each training fold, the fitted Cox model generated a linear predictor for each patient. Because concordance calculations depend on the direction in which the risk score is coded, score orientation was standardized within each training fold so that higher values consistently represented higher predicted risk. Specifically, concordance was evaluated for the linear predictor and its negation using only the training data, and the orientation corresponding to higher training-set concordance was retained. This fixed orientation was then applied to the held-out patient when generating the out-of-fold prediction. Because the orientation decision was based exclusively on the training data within each fold, no information from the held-out patient was used in this step.

Out-of-fold predictions were aggregated across all folds, and model performance was evaluated using Harrell’s concordance index [16]. Before pooling, risk-score direction was standardized within each training fold so that higher scores consistently represented higher predicted risk. The resulting out-of-fold predictions were then pooled across patients to calculate a single cross-validated C-index for each modeling strategy. Because each patient’s prediction was generated from a model fit without that patient, the pooled concordance reflects discrimination based on out-of-fold predictions rather than apparent performance from the training data.

For Harrell’s concordance index, score scale is not relevant because the statistic depends only on the ranking of predicted risks; fold-level orientation ensured consistent risk direction. For the Kaplan–Meier analyses, pooled out-of-fold risk scores were dichotomized at their median without additional normalization. Given the extensive overlap between LOOCV training sets (22 of 23 patients), along with predictor standardization and ridge penalization, substantial between-fold differences in score scale were not expected, although minor variation is inherent to cross-validation.

**Figure A1:**
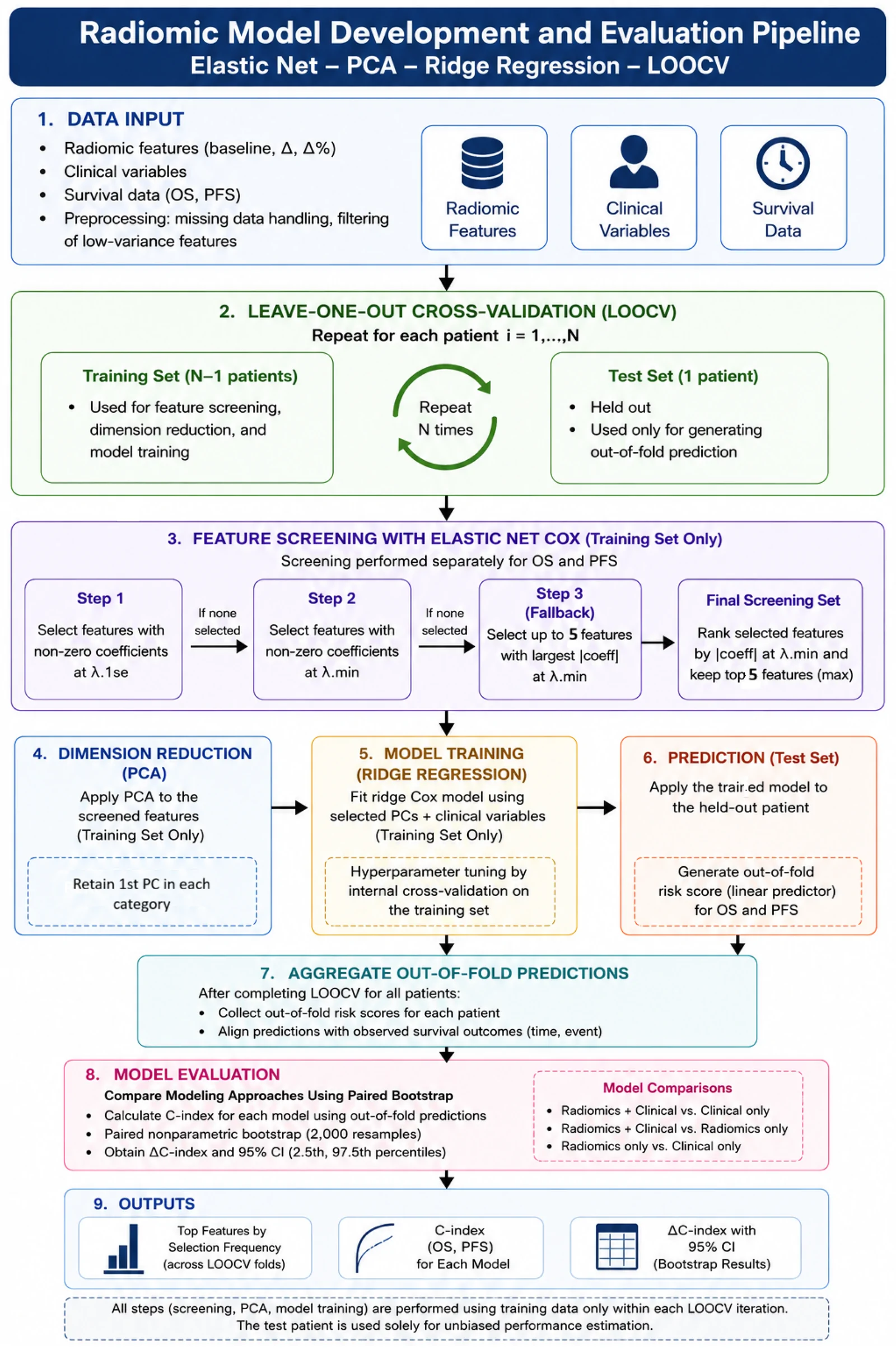
Flowchart of analysis pipeline.

Bootstrap resampling [17] was used to compare out-of-fold model discrimination between modeling strategies. Differences in concordance indices (ΔC-index) were estimated using a paired nonparametric bootstrap procedure. Out-of-fold predictions from the two models were matched by observation identifier, and the observed C-index was calculated separately for each model, with ΔC-index defined as the C-index for the first model minus that for the comparison model. We generated 2000 bootstrap samples by resampling observations with replacement, retaining the paired predictions from both models within each sampled observation. The C-index for each model and their difference were recalculated in every bootstrap sample, and the 95% confidence interval was obtained from the 2.5th and 97.5th percentiles of the resulting bootstrap distribution. A fixed random seed was used to ensure reproducibility. Because the models were evaluated using the same resampled observations, this paired approach accounts for the correlation between their C-index estimates. The bootstrap resampled the pooled out-of-fold predictions rather than repeating the full modeling pipeline within each resample; the resulting confidence intervals therefore reflect uncertainty in the concordance comparison given fixed out-of-fold predictions, and do not propagate variability from the feature-selection, dimensionality-reduction, or model-fitting steps. Pairwise comparisons were performed for the combined radiomics and clinical model versus the clinical-only model, the combined model versus the radiomics-only model, and the radiomics-only model versus the clinical-only model. These analyses were used to descriptively assess relative model performance rather than to provide definitive hypothesis testing, given the small sample size. This structured approach was selected to balance feature selection, dimensionality reduction, and shrinkage while mitigating overfitting in a small-sample, high-dimensional setting. All analysis was done in R v4.5.3. A detailed flowchart of the analysis pipeline is provided in Appendix A (Figure A1).

### 2.3. Landmark Analysis

Because delta radiomic features require a two-month follow-up scan, patients must have survived to that time point before their radiomic data could be calculated. This creates a potential guarantee-time bias in the primary analysis: patients who die or progress before the follow-up scan are necessarily excluded, and survival times are measured from study entry rather than from the time at which the radiomic data became available. To address this, a landmark analysis was conducted as a sensitivity analysis. The landmark time was set at two months from study entry, corresponding to the timing of the follow-up CT scan used for delta feature calculation. Only patients who remained at risk at the landmark time were included (*n* = 17), and survival times for both overall survival and progression-free survival were measured from the landmark time rather than from study entry. The same LOOCV modeling framework described above was then applied to this reduced cohort. The landmark analysis therefore provides a sensitivity assessment of model discrimination among patients remaining at risk at two months while reducing potential guarantee-time bias in the primary analysis.

## 3. Results

### 3.1. Patient Characteristics

The analytic cohort consisted of 23 patients with advanced NSCLC. The median age was 68 years, and the majority of patients were male. Most patients had lung lesions at baseline, with a subset demonstrating lymph node involvement. Clinical response distributions indicated that stable disease was the most common outcome, with smaller proportions of partial response and progressive disease (Table 1).

### 3.2. Model Performance

Model performance varied across the three modeling strategies. The clinical-only model demonstrated modest predictive performance, with concordance indices of 0.605 for overall survival and 0.571 for progression-free survival. The radiomics-only model showed similar performance for overall survival (C-index 0.609) but substantially improved discrimination for progression-free survival (C-index 0.770). The combined radiomics and clinical model achieved the best performance for overall survival (C-index 0.648) while maintaining favorable performance for progression-free survival (C-index 0.718) (Table 2).

Given the small cohort size, the clinical candidate set was intentionally limited to age at study entry and two baseline lesion-location indicators: the presence of lung lesions and lymph node involvement. These variables were consistently available and were used as parsimonious measures of baseline disease distribution and burden.

**Figure 1:**
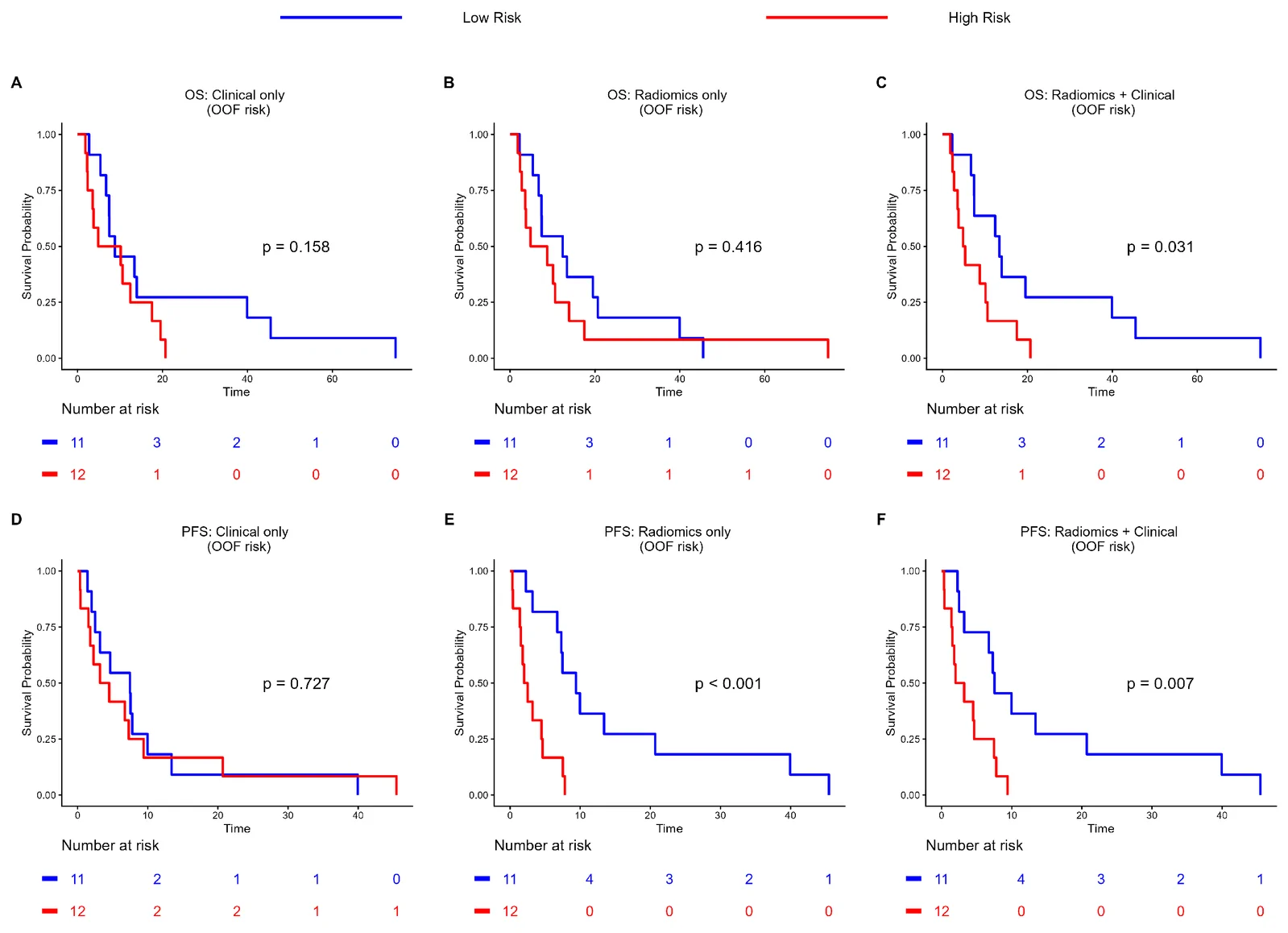
Kaplan–Meier curves for OS and PFS stratified by median out-of-fold risk scores. Risk groups were derived from leave-one-out cross-validated models. Log-rank *p*-values and numbers at risk are shown. (**A**) Overall Survival (OS): Clinical-only model; (**B**) Overall Survival (OS): Radiomics-only model; (**C**) Overall Survival (OS): Radiomics + Clinical model; (**D**) Progression-Free Survival (PFS): Clinical-only model; (**E**) Progression-Free Survival (PFS): Radiomics-only model; (**F**) Progression-Free Survival (PFS): Radiomics + Clinical model.

Kaplan–Meier [18] analyses based on median splits of out-of-fold risk scores demonstrated improved separation between high- and low-risk groups for the combined model, particularly for overall survival. Radiomics-only models showed notable separation for progression-free survival, whereas clinical-only models showed limited discrimination (Figure 1).

For overall survival (OS), the combined radiomics and clinical model showed the strongest separation between high- and low-risk groups (log-rank [19] *p* = 0.03). In contrast, the radiomics-only model did not demonstrate meaningful separation (*p* = 0.42), and the clinical-only model showed limited separation between risk groups (*p* = 0.16). These findings suggest that integration of clinical variables with radiomic features may improve stratification of OS risk.

For progression-free survival (PFS), the radiomics-only model demonstrated the most pronounced separation between risk groups (*p* < 0.001). The combined radiomics and clinical model also showed notable separation (*p* = 0.007), though less extreme than the radiomics-only model. The clinical-only model showed little separation between the dichotomized risk groups (*p* = 0.73).

Across both endpoints, the KM curves for the radiomics-based models exhibited earlier and more sustained divergence between risk groups, particularly for PFS, indicating that radiomic features capture early disease dynamics associated with progression. In contrast, clinical variables alone provided limited stratification.

Notably, the relative performance of models differed between KM-based group separation and concordance index estimates. While the radiomics-only model achieved the highest concordance for PFS, the combined model demonstrated improved separation for OS in KM analyses. This reflects the distinction between continuous risk discrimination and dichotomized risk stratification, which may emphasize different aspects of model performance.

Because six log-rank tests were conducted across three modeling approaches and two survival endpoints, we adjusted the nominal *p*-values using the Holm procedure [20] to control the family-wise error rate. After adjustment, the combined radiomics and clinical model was no longer statistically significant for OS (unadjusted *p* = 0.0315; Holm-adjusted *p* = 0.126). In contrast, the radiomics-only model for PFS remained statistically significant (unadjusted *p* = 0.000831; Holm-adjusted *p* = 0.00499), as did the combined radiomics and clinical model for PFS (unadjusted *p* = 0.00711; Holm-adjusted *p* = 0.0355). The remaining comparisons were not statistically significant after adjustment. These findings indicate that the apparent OS separation should be interpreted cautiously, whereas the PFS separation observed for the radiomics-based models was more robust to correction for multiple testing.

### 3.3. Cross-Validation Feature Selection Frequency and Biological Context

Cross-validation feature selection frequency identified a subset of radiomic features consistently selected across cross-validation folds. These features were predominantly texture-based and included Laws filter features, co-occurrence matrix features, and measures of intensity variation and tumor shape. The most frequently selected features reflected spatial heterogeneity and structural complexity, supporting their biological relevance in tumor characterization (Table 3).

Feature-selection sensitivity analyses using elastic-net mixing parameters of α=0.25 and α=0.75 showed that the principal findings were generally robust. Maximum histogram gradient was selected in all 23 folds under each setting, while the Laws texture feature f241_3d_laws_features_r5_s5_s5 was selected in 23, 23, and 22 folds for α=0.25, 0.50, and 0.75, respectively. Several first-order and co-occurrence features also showed similar selection frequencies across settings. Some secondary features were more sensitive to the mixing parameter. Major-axis length increased in frequency from 12 to 18 to 21 folds as α increased, whereas coefficient of variation decreased from 16 to 12 to 9 folds and the 90th-percentile intensity feature decreased from 16 to 9 to 8 folds. Overall, the core selected feature set was stable, although the relative ranking of several secondary features varied with the penalty mixture.

Sensitivity analyses also examined the effect of the mixing parameter on overall model discrimination. The clinical-only model was unaffected across all three settings, as clinical variables are not subject to elastic-net screening. For radiomic-based models, α=0.75 produced results nearly identical to the primary analysis, with absolute changes in C-index of ≤0.024 across all model–endpoint combinations. The α=0.25 setting yielded modestly higher C-indices, with the largest differences observed for the radiomics-only OS model (ΔC-index = +0.067) and the combined radiomics and clinical PFS model (ΔC-index = +0.075). These findings indicate that model discrimination was somewhat higher under the more ridge-like α=0.25 screening setting, while the overall discrimination pattern was generally robust to the choice of penalty mixture.

Several recent studies provide evidence that radiomic texture features reflect biologically meaningful characteristics of tumor architecture and the tumor microenvironment. In the landmark radiogenomics study by Aerts et al. [4], radiomic features suggested associations with gene-expression patterns related to cell cycling, proliferation, and other tumor biological processes, providing preliminary evidence of a link between imaging phenotype and underlying molecular characteristics. More recently, Huang et al. [21] reported associations between radiomic risk profiles, tumor-related signaling pathways, and immune microenvironment characteristics in NSCLC. These findings support the interpretation that spatial heterogeneity quantified by CT texture features may correspond to measurable differences in tumor molecular biology and immune composition.

In NSCLC specifically, Yoon et al. [22] demonstrated that CT-derived radiomic features were associated with immune-cell infiltration and tumor microenvironment profiles, while Guo et al. [23] identified CT radiomic subtypes associated with immunotherapy efficacy and microenvironmental characteristics across multiple cohorts. Lee et al. [24] further showed that radiomic features in lung adenocarcinoma were associated with prognostic and pathological characteristics, including measures reflecting tumor size, intensity distribution, and intratumoral heterogeneity. Coroller et al. [25] demonstrated that pretreatment radiomic phenotypes predicted pathological response to neoadjuvant chemoradiation in NSCLC, providing an additional link between imaging-derived characteristics and tissue-level treatment effects. A recent systematic review and meta-analysis by Zhang et al. [26] confirmed that pretreatment CT texture features can stratify survival outcomes in patients with advanced NSCLC receiving immunotherapy. Similarly, Deng et al. [27] reported that radiomic signatures associated with tumor immune heterogeneity predicted survival in patients with brain metastases from NSCLC. Collectively, these studies reinforce the prognostic and biological relevance of imaging-derived heterogeneity measures.

Particularly relevant to our longitudinal approach, Liu et al. [28] used serial CT radiomics to non-invasively characterize the tumor immune microenvironment and predict immunotherapy response in lung cancer, suggesting that temporal changes in radiomic features may capture dynamic immune–tumor interactions. Bernatowicz et al. [29] similarly developed a radiomic signature for longitudinal monitoring of the tumor-inflamed microenvironment and immunotherapy response. More broadly, recent work on habitat imaging has emphasized the value of characterizing spatially distinct tumor regions to better represent intratumoral heterogeneity [30]. These studies support the biological plausibility of our delta-radiomic approach, in which early changes in texture and co-occurrence features may reflect treatment-related remodeling of tumor architecture, changes in cellular density or necrosis, and alterations in immune microenvironment composition. Taken together, this body of evidence supports the interpretation that the Laws filter, GLCM, and run-length features frequently selected in our cross-validation framework may capture biologically relevant spatial heterogeneity rather than imaging noise alone.

### 3.4. Bootstrap Comparisons

For OS, the combined radiomics and clinical model showed numerically higher discrimination than the clinical-only model (ΔC-index = 0.044; 95% CI, −0.152 to 0.232) and the radiomics-only model (ΔC-index = 0.040; 95% CI, −0.074 to 0.157). The radiomics-only model showed minimal difference from the clinical-only model for OS (ΔC-index = 0.004; 95% CI, −0.219 to 0.227). For PFS, the combined model showed numerically higher discrimination than the clinical-only model (ΔC-index = 0.147; 95% CI, −0.074 to 0.339), whereas the radiomics-only model had the largest observed improvement over the clinical-only model (ΔC-index = 0.198; 95% CI, −0.013 to 0.397). The combined model showed slightly lower discrimination than the radiomics-only model for PFS (ΔC-index = −0.052; 95% CI, −0.167 to 0.051). However, all bootstrap confidence intervals crossed zero, indicating that these differences were not statistically distinguishable in this small cohort. Therefore, these comparisons should be interpreted as exploratory evidence of relative model performance rather than confirmatory evidence of superiority (Table 4).

### 3.5. Landmark Analysis Sensitivity

In this small cohort (*n* = 23), the model demonstrated moderate discrimination in the conventional time-to-event analysis, with a C-index of 0.718 for PFS in the Radiomics + Clinical model. However, model performance was attenuated after applying a landmark analysis (*n* = 17; 26% reduction in sample size), with the C-index decreasing to 0.639 for PFS and decreasing from 0.648 to 0.529 for OS. This reduction is not unexpected, as landmark analysis restricts the evaluable risk set to patients who remain at risk at the landmark time and reduces potential time-related or guarantee-time bias. Given the reduced sample size and conditional risk set, the landmark estimate should be interpreted cautiously as a complementary sensitivity analysis rather than directly compared as a less biased estimate of the primary C-index.

## 4. Discussion

This study demonstrates that early changes in radiomic features capturing tumor heterogeneity are associated with survival outcomes in advanced NSCLC. The results highlight the importance of incorporating longitudinal imaging information and integrating radiomic features with clinical variables within a rigorous statistical framework. Given the small sample size and absence of external validation, these findings should be considered exploratory and hypothesis-generating.

Radiomics-only models showed favorable performance for progression-free survival, suggesting that imaging-derived measures capture early biological changes associated with disease progression. In contrast, the addition of clinical variables was associated with numerically higher discrimination for overall survival, indicating that patient-level factors contribute complementary prognostic information beyond imaging-derived heterogeneity metrics.

The prominence of texture and filter-based features among stable predictors underscores the importance of spatial heterogeneity in tumor biology. To provide biological context for the radiomic features most frequently selected across cross-validation folds, we mapped representative feature classes to their underlying quantitative properties and plausible biological interpretations (Table 5). Texture-based features, including those derived from Laws filters and gray-level co-occurrence matrices (GLCMz), quantify local structural patterns, intensity heterogeneity, and spatial organization within the tumor. These characteristics are thought to reflect micro-architectural complexity and intratumoral heterogeneity, potentially capturing variability in cellular density, necrosis, and microenvironmental composition. Similarly, run-length and size-zone features characterize the extent and continuity of homogeneous intensity regions, which may reflect disruption of uniform tissue architecture associated with treatment-induced remodeling. Taken together, these feature classes highlight the capacity of radiomics to capture biologically meaningful spatial heterogeneity that may not be discernible through conventional imaging metrics alone. These observations are consistent with prior work linking radiomic heterogeneity to clinical outcomes and further support the biological relevance of imaging-derived texture features.

The modeling framework used in this study addresses several key challenges in radiomics research. By enforcing strict cross-validation, all preprocessing, feature selection, and model fitting steps were restricted to training data within each fold, minimizing information leakage and providing out-of-fold estimates of predictive performance [31]. The use of outcome-guided feature screening, category-aware dimensionality reduction, and penalized regression allowed for structured handling of high-dimensional, correlated radiomic features in a small sample setting. Sensitivity analyses using elastic-net mixing parameters of α=0.25, 0.50, and 0.75 showed that the highest-frequency features were largely preserved across settings, supporting the robustness of the core radiomic signal. However, changes in the selection frequencies and rankings of several secondary features indicate that some feature-level results remained sensitive to the penalty mixture, as expected in a small, highly correlated dataset.

Several limitations should be emphasized. The most important limitation is the extremely small sample size relative to the high dimensionality of the radiomic feature space. Although 309 radiomic features were initially available, the analytic cohort included only 23 patients. This imbalance substantially increases the risk that selected features and apparent predictive patterns reflect sampling variation rather than reproducible biological associations. Even when penalized regression, dimensionality reduction, and strict cross-validation are used, small changes in the composition of the training sample can alter which features are selected, the estimated principal components, and the fitted model coefficients. The cohort was also racially homogeneous, with more than 90% of participants identified as White, which limits the ability to assess whether the observed radiomic associations and model performance generalize across more diverse patient populations [32].

Although noninformative features with zero or nonfinite variance were excluded within the cross-validation pipeline, this procedure does not establish measurement reproducibility. Features were not prescreened using test–retest imaging or repeated-segmentation reliability criteria; consequently, some selected features may be sensitive to acquisition or segmentation variability. Future studies should incorporate formal reproducibility assessment and restrict model development to features demonstrating acceptable reliability [33].

The clinical-only comparator was based on a restricted set of variables consisting of age and baseline lung and lymph node involvement. Although lesion-location indicators provide information regarding the anatomic distribution of disease, they do not capture the full range of established prognostic factors in advanced NSCLC. In particular, PD-L1 status and ECOG performance status were unavailable. Consequently, the clinical-only model may underestimate the predictive performance achievable with a more comprehensive clinical dataset, and apparent advantages of radiomics should be interpreted cautiously.

Additional limitations relate to imaging acquisition and segmentation. CT scans were acquired on Siemens and GE scanners using standard-of-care protocols with variation in slice thickness and pixel spacing. Image intensities were discretized using a fixed bin width of 25 Hounsfield units, but no voxel resampling, interpolation, or scanner harmonization was performed. Consequently, some radiomic variation may reflect scanner or acquisition differences rather than biological change, and the small cohort prevented reliable adjustment for these potential confounders. In addition, formal intraobserver and interobserver reproducibility analyses were not performed. Although segmentations were completed by an experienced imaging scientist using a standardized semiautomated method and approximately 10% were reviewed by a radiologist for quality assurance, repeated segmentations were unavailable. Therefore, the sensitivity of selected features to observer-related segmentation variability could not be quantified. Future studies should incorporate standardized acquisition or resampling procedures, evaluate harmonization approaches, and assess feature reproducibility using repeated segmentations and intraclass correlation coefficients.

Consequently, the observed feature-selection frequencies, risk scores, and concordance estimates may be highly sensitive to individual patients and should not be interpreted as evidence of a stable or definitive radiomic signature. The small number of patients also limits the ability to estimate model performance precisely, evaluate interactions or nonlinear effects, include a broader set of clinical covariates, or assess calibration. Therefore, the reported C-indices and Kaplan–Meier separations should be regarded as exploratory estimates that may be optimistic and require confirmation in a substantially larger independent cohort.

Leave-one-out cross-validation, while efficient in small datasets, may produce high-variance estimates and is sensitive to individual observations. As an additional diagnostic, within-training-fold C-indices used for risk-score orientation were consistently higher than the pooled out-of-fold C-indices reported as model performance. Across folds, selected training concordance ranged from 0.630 to 0.712 for OS and 0.707 to 0.776 for PFS in the clinical-only model, from 0.732 to 0.835 for OS and 0.822 to 0.896 for PFS in the radiomics-only model, and from 0.745 to 0.823 for OS and 0.835 to 0.909 for PFS in the combined model. These values reflect within-training-fold fit rather than validated predictive performance and further emphasize the potential optimism and instability expected in a small LOOCV analysis. Because feature selection is outcome-guided within each fold, selected features may vary across folds, reflecting instability inherent to small-sample radiomics analyses. Additionally, the absence of external validation limits the generalizability of these findings, and validation in independent cohorts is essential to confirm their robustness [32]. Finally, while dimensionality reduction improves model stability, the use of principal components limits interpretability of individual radiomic features and may hinder direct biological interpretation.

The proportional hazards assumption was not formally assessed. Within the LOOCV framework, a separate penalized Cox model was fit in each of the 23 training folds, each with its own selected features, principal components, and estimated coefficients. Because the analysis was focused on risk prediction and discrimination rather than inference on individual coefficients or hazard ratios, fold-specific proportional hazards diagnostics were not considered informative in this small-sample setting. Model performance was evaluated using the concordance index, a rank-based measure of discrimination that does not itself require the proportional hazards assumption to hold.

A further methodological consideration concerns the comparability of fold-specific out-of-fold risk scores when pooled across LOOCV folds. Because each fold’s model may select different features, estimate distinct principal components, and apply different penalty parameters, the resulting linear predictors could differ in scale across folds. To address this, we conducted a fold-normalization sensitivity analysis in which each patient’s held-out linear predictor was re-expressed in units of the training-fold standard deviation above the training-fold mean linear predictor (fold-z-normalization), with the same global orientation logic applied as in the primary analysis. In summary, fold-normalized C-indices were broadly consistent with primary estimates: the radiomics-only model remained the strongest for PFS (fold-normalized C-index 0.794 vs. primary 0.770), the combined model showed improved PFS discrimination under fold-normalization (0.770 vs. primary 0.718), and OS estimates were broadly preserved across all models. Kaplan–Meier analyses based on fold-normalized median splits produced qualitatively consistent findings. One notable discrepancy was observed for the clinical-only PFS C-index, which increased from 0.571 (primary) to 0.698 (fold-normalized), suggesting that the magnitude of the apparent radiomic advantage over clinical variables for PFS may be partly influenced by between-fold score-scale differences. Even accounting for this, the radiomics-only model substantially outperformed the clinical-only model under fold-normalization (0.794 vs. 0.698), and the qualitative conclusions of the primary analysis are preserved. Nonetheless, the fold-scale artifact in the clinical-only PFS estimate reinforces the exploratory nature of the reported results and the need for independent external validation.

Notably, differences in model performance between concordance index estimates and Kaplan–Meier stratification highlight that continuous risk discrimination and dichotomized risk grouping capture distinct aspects of predictive performance. While radiomics-only models achieved the highest concordance for progression-free survival, combined models demonstrated improved separation for overall survival following dichotomization, suggesting that integration of clinical variables may enhance risk stratification in certain contexts.

## 5. Conclusions

This study demonstrates the feasibility of integrating longitudinal radiomic features within a structured survival modeling framework to investigate imaging biomarkers in a small early-phase clinical trial. By combining outcome-guided feature screening, category-aware dimensionality reduction, and penalized Cox modeling within a strict leave-one-out cross-validation framework, the analysis was designed to limit overfitting while maximizing the use of limited data.

Across modeling approaches, radiomic features capturing spatial heterogeneity—particularly texture-based metrics derived from Laws filters and gray-level co-occurrence matrices—were consistently selected and showed associations with survival outcomes. These findings suggest that longitudinal changes in tumor heterogeneity may reflect biologically relevant dynamics during treatment, particularly for progression-free survival.

However, given the small sample size and absence of external validation, these results should be considered exploratory and hypothesis-generating. Model performance estimates are sensitive to individual observations, and independent validation in larger cohorts will be necessary to confirm the robustness and generalizability of these findings.

Overall, this work highlights the potential role of longitudinal radiomic features as candidate imaging biomarkers in advanced NSCLC and provides a structured analytical framework for studying high-dimensional radiomic data in small clinical datasets. If validated, such imaging-based risk stratification approaches could inform early treatment decision-making or patient monitoring strategies. Despite the use of internal cross-validation, the findings require confirmation in an independent external cohort before the proposed radiomic models can be considered generalizable or suitable for clinical use.

## Figures and Tables

**Table 1:** Overall descriptive statistics for the analytic cohort.

Characteristic	Level	Overall, N = 23
Best response	Partial Response	3 (13.0%)
	Progressive Disease	6 (26.1%)
	Stable Disease	14 (60.9%)
Disease control	DC	17 (73.9%)
	PD	6 (26.1%)
Race	Asian	1 (4.35%)
	White	21 (91.3%)
	White; Unknown	1 (4.35%)
Biological sex	Female	7 (30.4%)
	Male	16 (69.6%)
Age at study entry	Median [range]	68.0 [38.0; 82.0]
Site of lesion at baseline	Adrenal Glands	3 (13.0%)
	Liver	3 (13.0%)
	Lung & Bronchus	10 (43.5%)
	Lymph Nodes	7 (30.4%)

Categorical variables are summarized as n (%). Age is summarized as median [range]. Site of lesion at baseline represents the site of that patient’s segmented lesions.

**Table 2:** Model performance based on pooled out-of-fold C-index for OS and PFS.

Model	OS C-Index	PFS C-Index
Clinical only	0.605	0.571
Radiomics only	0.609	0.770
Radiomics + Clinical	0.648	0.718

**Table 3:** Top radiomic features by elastic-net screening frequency across 23 LOOCV folds. Union = OS ∪ PFS; frequencies reflect the combined radiomics and clinical model.

Feature	Union	PFS	OS
f241_3d_laws_features_r5_s5_s5	23	23	4
f39_maximum_histogram_gradient	23	23	3
f61_major_axis_length	18	18	0
f1_statistical_mean	14	0	14
f10_statistical_maximum_grey_level	14	0	14
f100_avg_coocurrence_sum_variance	14	0	14
f101_avg_coocurrence_sum_entropy	14	0	14
f102_avg_coocurrence_angular_second_moment	14	0	14
f16_statistical_coefficient_of_variance	12	12	0
f199_3d_laws_features_e5_w5_l5	9	9	2
f9_statistical_90th_percentile	9	7	2
f295_3d_wavelet_p1_l2_c9	7	0	7
f243_3d_laws_features_r5_s5_w5	5	5	2
f268_3d_laws_features_w5_s5_w5	5	1	4
f283_3d_wavelet_p1_l2_c3	5	0	5

**Table 4:** Bootstrap differences in C-index between modeling approaches.

Endpoint	Comparison	ΔC-Index	95% CI
	Radiomics + Clinical vs. Clinical only	0.044	(−0.152, 0.232)
OS	Radiomics + Clinical vs. Radiomics only	0.040	(−0.074, 0.157)
	Radiomics only vs. Clinical only	0.004	(−0.219, 0.227)
	Radiomics + Clinical vs. Clinical only	0.147	(−0.074, 0.339)
PFS	Radiomics + Clinical vs. Radiomics only	−0.052	(−0.167, 0.051)
	Radiomics only vs. Clinical only	0.198	(−0.013, 0.397)

**Table 5:** Radiomic feature classes and biological interpretation.

Feature Class	What It Measures	Plausible Biological Interpretation
Laws texture filters	Local structural patterns and edge-like intensity variations	Micro-architectural complexity and heterogeneous tissue organization
GLCM entropy/contrast	Spatial variability and intensity dissimilarity	Intratumoral heterogeneity, including mixed viable and necrotic regions
Run length/zone features	Size and continuity of homogeneous regions	Breakdown of uniform tissue due to treatment-induced remodeling

## Data Availability

All data generated in this study are available from the corresponding authors upon reasonable request.

## Abbreviations

The following abbreviations are used in this manuscript:

NSCLC	Non-small cell lung cancer
LOOCV	Leave one-out cross-validation
OS	Overall survival
PFS	Progression free survival
GLCM	Gray-level Co-occurrence Matrix
GLRLM	Gray-level Run Length Matrix
GLSZM	Gray-level Size Zone Matrix
RECIST	Response Evaluation Criteria in Solid Tumors
CT	Computed Tomography
TMWs	Tumor measurement worksheets
KM	Kaplan–Meier

## Appendix B

**Table A1:** R packages and versions used in the analysis.

Package	Version	Date	Source
broom	1.0.12	27 January 2026	CRAN (R 4.5.3)
compareGroups	4.10.2	8 January 2026	CRAN (R 4.5.3)
dplyr	1.2.1	3 April 2026	CRAN (R 4.5.3)
forcats	1.0.1	25 September 2025	CRAN (R 4.5.3)
fs	2.0.1	24 March 2026	CRAN (R 4.5.3)
ggbreak	0.1.7	18 March 2026	CRAN (R 4.5.3)
ggiraph	0.9.6	21 February 2026	CRAN (R 4.5.3)
ggplot2	4.0.3	22 April 2026	CRAN (R 4.5.3)
ggpubr	0.6.3	24 February 2026	CRAN (R 4.5.3)
glmnet	4.1–10	17 July 2025	CRAN (R 4.5.3)
gt	1.3.0	22 January 2026	CRAN (R 4.5.3)
gtExtras	0.6.2	17 January 2026	CRAN (R 4.5.3)
gtsummary	2.5.0	5 December 2025	CRAN (R 4.5.3)
kableExtra	1.4.0	24 January 2024	CRAN (R 4.5.3)
knitr	1.51	20 December 2025	CRAN (R 4.5.3)
lubridate	1.9.5	4 February 2026	CRAN (R 4.5.3)
MASS	7.3–65	28 February 2025	CRAN (R 4.5.3)
Matrix	1.7–4	28 August 2025	CRAN (R 4.5.3)
patchwork	1.3.2	25 August 2025	CRAN (R 4.5.3)
purrr	1.2.2	10 April 2026	CRAN (R 4.5.3)
quarto	1.5.1	4 September 2025	CRAN (R 4.5.3)
reactable	0.4.5	1 December 2025	CRAN (R 4.5.3)
readr	2.2.0	19 February 2026	CRAN (R 4.5.3)
rmarkdown	2.31	26 March 2026	CRAN (R 4.5.3)
skimr	2.2.2	10 January 2026	CRAN (R 4.5.3)
stringr	1.6.0	4 November 2025	CRAN (R 4.5.3)
survival	3.8–6	16 January 2026	CRAN (R 4.5.3)
survminer	0.5.2	25 February 2026	CRAN (R 4.5.3)
tibble	3.3.1	11 January 2026	CRAN (R 4.5.3)
tidyr	1.3.2	19 December 2025	CRAN (R 4.5.3)
tidyverse	2.0.0	22 February 2023	CRAN (R 4.5.3)
